# Novel MR-Guided Radiotherapy Elective Rotation for Radiation Oncology Trainees

**DOI:** 10.7759/cureus.10706

**Published:** 2020-09-29

**Authors:** Austin J Sim, Jessica M Frakes, Sarah E Hoffe, Evan Wuthrick, Thomas J Dilling, Stephen Rosenberg

**Affiliations:** 1 Radiation Oncology, Moffitt Cancer Center, Tampa, USA

**Keywords:** magnetic resonance imaging-guided radiation therapy (mrigrt), stereotactic body radiation therapy (sbrt), adaptive radiation therapy, graduate medical education

## Abstract

MR-guided adaptive radiation therapy (RT) is emerging as an integral treatment modality for certain applications and is poised to become an exciting opportunity for greater treatment precision and personalization. However, this is still a relatively nascent technology and only a few institutions and programs have access to this technology for clinical use and trainee education. To increase the diversity of elective offerings and improve the understanding of an MR-guided radiotherapy program, we initiated a unique MR-guided radiotherapy elective rotation for radiation oncology residents. During a representative four-week rotation, 21 simulations were completed by the resident on service. A plurality of simulations were for pancreas stereotactic body radiation therapy (SBRT; 48%) and a majority (71%) of simulations were for adaptive treatments. Additionally, 74 adaptive fractions were completed during this month, of which a significant majority (74%) were for pancreas SBRT. Of the non-adaptive fractions, the majority were for prostate SBRT and intensity-modulated radiation therapy (IMRT). Although many programs may offer training in some aspects of MR-guided radiotherapy as trainees rotate through certain disease sites, we hope this may serve as a blueprint to encourage programs with this technology to fully embrace training in essential competencies related to MR-guided radiotherapy. MR-guided radiotherapy has unique challenges that trainees need to understand to deliver treatment safely: geometric uncertainty, MRI to RT isocenter, and uncertainties with voxel size/tracking.

## Introduction

MR-guided adaptive radiation therapy (RT) is emerging as an integral treatment modality for certain applications and is poised to become an exciting opportunity for greater treatment precision and personalization. The improved soft tissue contrast and opportunities for real-time, intrafraction tracking allow for the reduction of planning target volume (PTV) by decreasing tumor and organ at risk uncertainty [1]. The emergence of radiomic biomarkers, use of multiple imaging sequences, offers unique opportunities to see day-to-day changes in radiomic features to develop novel biomarkers to assess for tumor response, as has already started in diagnostic functional imaging [2]. Two commercial systems are FDA approved and are in routine clinical use in the United States, with many more systems under development across the globe [3]. However, this is still a relatively nascent technology and only a few institutions and programs have access to this technology for clinical use and trainee education. We have previously examined a lack of MRI familiarity among trainees in radiation oncology [4]. In that context, we have worked to develop an MR-guided elective to improve the understanding of this technology for residents in our program.

An MR-linac has been in routine clinical use at our institution (Moffitt Cancer Center, Tampa, FL) since February 2019. We have completed over 250 MR-guided simulations in those 16 months with approximately one-third of patients being treated with adaptive radiotherapy. On the MR-linac at our institution, the breakdown by treatment of disease site is 30%, pancreas; 20%, thoracic; 15%, prostate; 10%, liver, and mixture of other sites.

In the United States, residency in radiation oncology lasts for four years after completion of an intern year. After the completion of required rotations and up to 12 months of elective/research time, residents in our program typically have two or three additional months of clinical elective time. Residents are able to take this time to revisit disease sites or pursue new ones, like cutaneous. To increase the diversity of elective offerings and improve the understanding of MR-guided radiotherapy program, we initiated a unique MR-guided radiotherapy elective rotation for radiation oncology residents.

## Materials and methods

Data regarding number and types of simulations and adaptive treatments were collected for a single resident on a representative four-week rotation. Results are described descriptively.

## Results

This four-week rotation is designed for senior residents in their penultimate or final years (postgraduate year [PGY]-4 and PGY-5) who have completed rotations in core disease sites. During this elective, the resident covers all simulations on our MR-linac not already covered by another service and all adaptive fractions. Coverage of non-adaptive stereotactic body radiation therapy (SBRT) fractions is encouraged but not mandatory. Contours are completed by the resident and reviewed with the treating attending physician. During adaptive fractions, the resident is primarily responsible for confirming rigid shifts, recontouring critical structures based on the anatomy of the day, evaluating adaptive plans, and adapted plan quality assurance, with final plan approval by the attending physician and medical physicist.

**Figure 1:**
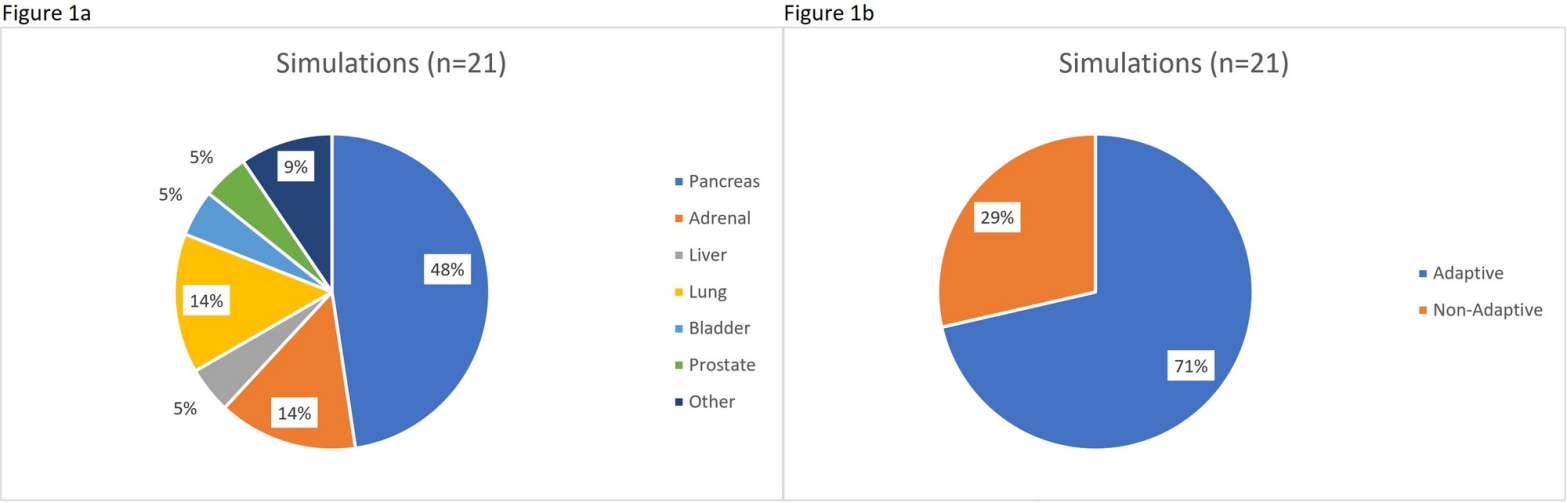
Distribution of simulations Breakdown of simulations during a representative month on the MR-guided rotation by (a) disease site and (b) adaptive versus non-adaptive fractions

**Figure 2:**
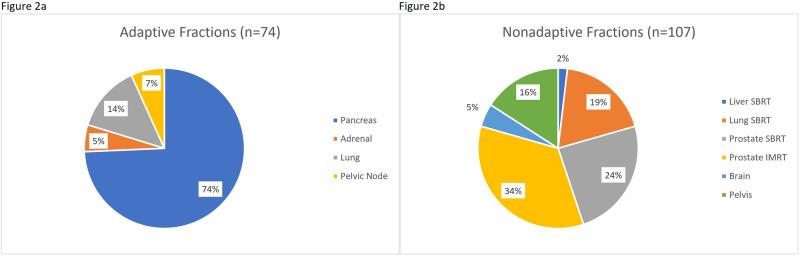
Distribution of treated fractions Breakdown of fractions treated by histology during a representative month on the MR-guided rotation comparing (a) adaptive fractions and (b) non-adaptive fractions. SBRT: stereotactic body radiation therapy; IMRT: intensity-modulated radiation therapy

During a representative four-week rotation, all simulations not covered by another resident were completed by the resident on service (n=21, an additional seven MR-guided prostate SBRT simulations were completed by the resident on the genitourinary service). A plurality of simulations were for pancreas SBRT (48%) and 71% of simulations were for adaptive treatments (Figures 1a, 1b). Seventy-four adaptive fractions were completed during this month, of which a significant majority (74%) were for pancreas SBRT. Of the non-adaptive fractions, the majority were for prostate SBRT and intensity-modulated radiation therapy (IMRT) (Figures 2a, 2b).

Expected competencies to be mastered by the trainee include (1) the indications and appropriate patient selection for online and offline MR-guided radiotherapy, (2) unique considerations of tracking structures at patient simulation, and (3) real-time re-planning for online adaptive radiotherapy (Table 1).

**Table 1:** MR-guided radiotherapy elective objectives PGY: postgraduate year; TRUFI: true fast imaging with steady-state precession; QA: quality assurance; ROI: region of interest; GTV: gross tumour volume; CTV: clinical target volume; PTV: planning target volume

PGY-4 objectives		PGY-5 objectives
Competency: Medical knowledge		
Goal: Clinical knowledge		
· Be able to determine which patients are eligible or ineligible for MR-guided radiotherapy.		· Understand treatment results and outcomes of MR-guided radiotherapy and be able to describe clinical trials (results and ongoing trials) and retrospective series.
· Fully understand indications for MR-guided radiotherapy including rarer entities across anatomical sites.		· Master acute and chronic effects of MR-guided radiation therapy across disease sites.
· Fully understand the indications for real-time (online) and offline adaptive MR-guided radiotherapy.		· Master delivery of real-time adaptive MR-guided radiotherapy (online and offline) across disease sites.
· Understand treatment results and outcomes of MR-guided radiotherapy across malignancies.		
· Fully understand advantageous and disadvantages to MR-guided radiotherapy.		
Goal: Medical physics knowledge		
· Apply concepts of medical physics as it relates to MR-guided radiotherapy.		· Thoroughly understand medical physics concepts for safe delivery of MR-guided radiation therapy.
· Understand and be able to describe TRUFI sequence and basic MRI physics.		
· Understand and describe the different re-optimization processes and QA process as part of real-time adaptive MR-guided radiotherapy.		
Competency: Patient care		
Goal: Patient care		
· Understand decisions on ROI and tracking as it relates to MR-guided radiation.		· Make independent decisions on ROI and tracking structures for MR-guided treatment.
· Understand the need for CT scans as part of MR-guided treatment and when CT scans may not be necessary.		· Master the real-time review of patient treatment plans as it pertains to adaptive MR-guided treatment and delivery (i.e., delivery of original plan or adaptive plan).
· Delineate all target volumes nearly independently (GTV, CTV, PTV) on MRI and CT.		
· Perform the real-time review of patient treatment plans as it pertains to adaptive MR-guided treatment and delivery (i.e., delivery of original plan or adaptive plan).		
· Plan and perform MR-guided radiotherapy with minimal faculty member assistance		· Be able to independently plan and perform MR-guided radiotherapy appropriately; conduct clinical research.

## Discussion

To our knowledge, this is the first reported MR-guided radiotherapy elective rotation to be offered for radiation oncology trainees. Comfort with MR-guidance and adaptive radiotherapy is becoming a sought-after skill, especially in the treatment of certain thoracic [5] and gastrointestinal malignancies [6]. The use of diagnostic MR imaging in staging, treatment planning, and surveillance is rapidly becoming integral to the standard of care of an increasing number of disease sites, necessitating a shift in resident education to inculcate facility in interpreting and using these images. Additionally, online adaptive radiotherapy presents new skills for trainees to master [7]. Much like the chief resident service described by Jeans et al. [8], we believe this unique curricular offering addresses a need as we look to continually modernize our educational curricula to account for new technologies.

Although many programs may offer training in some aspects of MR-guided radiotherapy as trainees rotate through certain disease sites, we hope this may serve as a blueprint to encourage programs with this technology to fully embrace training in essential competencies related to MR-guided radiotherapy. MR-guided radiotherapy has unique challenges that trainees need to understand to deliver treatment safely: geometric uncertainty, MRI to RT isocenter, and uncertainties with voxel size/tracking. These were reviewed throughout the rotation, which gives trainees the tools to develop confidence in understanding how to troubleshoot difficult cases and safety in MR-guided treatment.

## Conclusions

As MR-guided radiotherapy continues to expand throughout the United States and worldwide, it is poised to become an essential tool in the radiotherapy armamentarium. As efforts are underway to reform the educational curriculum in radiation oncology, inclusion of MR-guided and adaptive radiotherapy should be considered more formally in radiation oncology residency training.
